# A Spine Friendly “Backdoor Anterolateral Retroperitoneal Approach” for the Treatment of Giant Extraforaminal L5‐S1 Spinal Schwannoma: A Case Report

**DOI:** 10.1155/crnm/3243101

**Published:** 2026-04-17

**Authors:** Kodeeswaran M., Chiraag Hiran S., Jagadish Chandrabose, Srikala Prasad, Priyadarshan K. P., Arun Narindar, Lakshmi Narasimhan Ranganathan, Bipin Chaurasia, Ganesh P. S.

**Affiliations:** ^1^ Apollo First Med Hospitals, Chennai, India; ^2^ Neurosurgery Academy and Research Foundation, NARF, GKMC, Chennai, India; ^3^ Department of Neurosurgery, Government Kilpauk Medical College and Hospital, Kilpauk, Chennai, Tamil Nadu, India; ^4^ Government Kilpauk Medical College and Hospital, Kilpauk, Chennai, Tamil Nadu, India; ^5^ Department of Neurology, Sri Ramachandra Institute of Higher Education and Research, Porur, Chennai, Tamil Nadu, India, sriramachandra.edu.in; ^6^ Department of Neurosurgery, Neurosurgery Clinic, Birgunj, Nepal

**Keywords:** anterolateral retroperitoneal approach, backdoor approach, extraforaminal schwannoma

## Abstract

Schwannomas, or peripheral nerve sheath tumors, involving the retroperitoneal space are rare, accounting for approximately 3%–5% of all tumors, and most commonly arise from the eighth cranial nerve and spinal nerve roots. Extraforaminal retroperitoneal spinal schwannomas of the lumbosacral region are particularly uncommon and present unique surgical challenges due to their deep location and proximity to major neurovascular structures. Gross total resection remains the treatment of choice; however, conventional posterior and transparaspinal approaches often require bone removal and extensive paraspinal muscle dissection, potentially resulting in postoperative pain, paraspinal muscle morbidity, and spinal instability. We report the case of a 39‐year‐old female who presented with clinical and radiological features consistent with an extraforaminal retroperitoneal L5–S1 spinal schwannoma. After a thorough preoperative evaluation, the patient underwent complete tumor excision using a backdoor anterolateral retroperitoneal approach, selected to minimize tissue disruption while preserving spinal biomechanics. This approach provided a wider operative corridor with enhanced visualization of the lesion and adjacent neural structures, facilitating safe dissection and gross total resection through the intervertebral foramen without violation of posterior bony elements or excessive muscular stripping. The postoperative course was uneventful, and spinal stability was maintained. This case demonstrates that the backdoor anterolateral retroperitoneal approach is a safe and effective alternative for selected extraforaminal retroperitoneal lumbosacral schwannomas, enabling adequate exposure and complete excision while avoiding the biomechanical compromise commonly associated with traditional posterior approaches.

## 1. Introduction

Schwannomas, synonymously called as neurilemmomas or neurinomas, have a predilection more toward the vestibulocochlear or the spinal nerves. Retroperitoneal extension of spinal schwannomas represents a rare clinical entity, comprising approximately 3%–5% of reported cases. These tumors characteristically exhibit slow progression and a benign biological course, often producing minimal or nonspecific symptoms [1]. This case report presents a 39‐year‐old female patient with a large lateralized extraforaminal L5‐S1 spinal schwannoma on the left side. The treatment of choice for a spinal schwannoma is always gross total resection through various approaches, such as classic posterior approach, Wiltse’s approach, and a minimally destructive retroperitoneal approach, based on its extent and location. Amongst the approaches, the best and ideal procedure for our patient is the backdoor anterolateral retroperitoneal approach, as it provides a less disruptive surgery rather than the usual extensively destructive procedure, hampering the patient’s spine stability.

## 2. Case Presentation

A 39‐year‐old female patient came to the Neurosurgery Outpatient (OP) Department of Government Kilpauk Medical College and Hospital, Chennai, Tamil Nadu, India, and presented with an ambiguous low backache since 6 months and radicular pain in the left gluteal region since 2 months. The pain aggravated on long standing, bending forward, and exertion and relieved on rest and analgesics. She also developed a recent history of numbness and tingling on the lateral aspect of the left foot from the ankle to the little toe. She did not have any bowel and bladder dysfunction. She had no comorbidities or any past surgical history.

On physical examination, the straight leg raise (SLR) test was positive on the left side at 60°. On motor examination, muscle strength was assessed using the Medical Research Council (MRC) grading system. Power in the right upper and right lower limbs was full (5/5) across all major muscle groups. The left upper limb also demonstrated normal strength (5/5). In the left lower limb, muscle strength was preserved (5/5) at the hip (flexion, extension, abduction, and adduction), knee (flexion and extension), and ankle (plantarflexion and dorsiflexion). However, isolated weakness was noted in dorsiflexion of the left great toe (extensor hallucis longus), graded as 4‐/5. No other focal motor deficits were identified. She had reduced sensation on the lateral aspect of the left foot from the ankle to the little toe in comparison to the contralateral normal side. Her reflexes and tone were normal.

**FIGURE 1:**
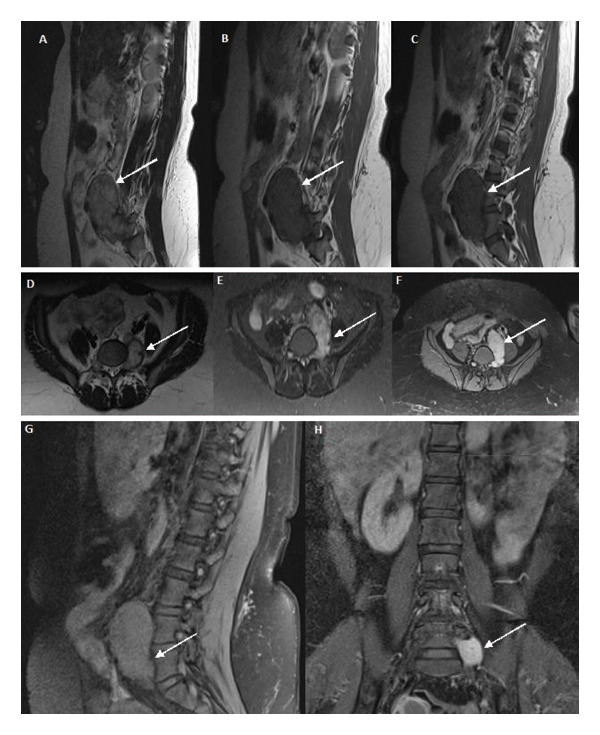
MRI spine (A), (B), (C) sagittal section; (D), (E), (F) axial section; (G) sagittal section; (H) coronal section. A 43 × 47 × 74mm well‐defined, T1‐isointense, T2‐hyperintense, lobulated lesion with homogeneous contrast enhancement, extending into the left L5–S1 foramen, and anterior to the left psoas muscle with anterior displacement of the left iliac vessels, suggestive of a left L5 spinal nerve sheath tumor or spinal schwannoma.

Based on the history and clinical findings mentioned above, a provisional diagnosis of spinal nerve compression was made. Hence, the patient was taken up for a contrast‐enhanced magnetic resonance imaging (MRI) of the lumbosacral spine with whole spine screening (Figure 1).

A multidisciplinary team meeting was arranged, and a decision of moving ahead with a backdoor anterolateral retroperitoneal approach was planned with the aim of providing a less destructive procedure in order to maintain spinal stability and remove the tumor in total.

**FIGURE 2:**
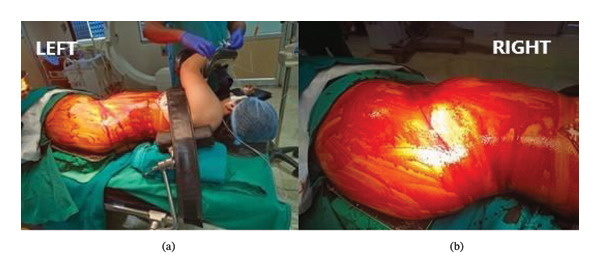
(a) Intraoperative right lateral decubitus position. (b) Left oblique lumbar incision.

The procedure was performed under general anesthesia. The patient was positioned in the right lateral decubitus position (Figure 2(a)). The operative field was prepared with 5% povidone–iodine solution and draped in a sterile manner.

An oblique incision (Figure 2(b)) over the left lumbar region measuring approximately 15 cm was placed. The incision extended laterally from the left iliac crest to the left subcostal margin and medially just above and parallel to the inguinal ligament.

**FIGURE 3:**
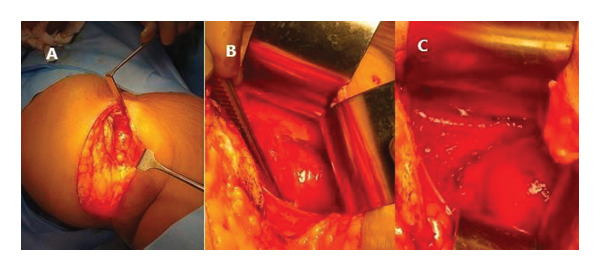
Intraoperative findings. (A) Lumbar oblique incision deepened along the layers of the lateral abdominal wall. (B) Retroperitoneal space created by retracting the peritoneum and its contents anteriorly. (C) Retroperitoneal space created and tumor head visualized.

The incision was deepened through the subcutaneous tissue. Sequential muscle dissection was carried out as follows:

1. External oblique muscle was divided in line with its fibers.
2. Internal oblique muscle was divided perpendicular to its fibers.
3. Transversus abdominis muscle and its fascia were divided in line with the skin incision (Figure 3(A)).

Upon identification of the peritoneum, blunt dissection was performed between the retroperitoneal fat and the psoas fascia. The peritoneum with its contents was gently retracted medially (Figure 3(B)).

The psoas muscle, ureter, and common iliac vessels were retracted laterally along with the peritoneal contents and retroperitoneal fat. The surface of the psoas muscle was traced to expose the lateral aspect of the L5–S1 vertebral body. The operative level was confirmed using a C‐arm fluoroscopy.

**FIGURE 4:**
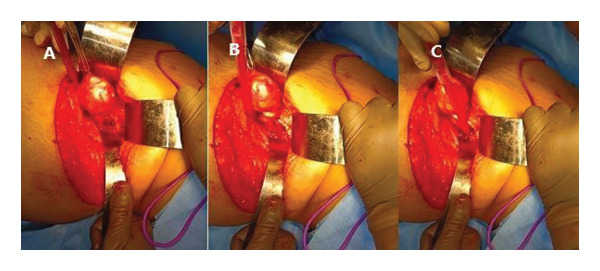
(A) Careful meticulous dissection done around the tumor base. (B) The tumor base found extending up to the intervertebral foramen of the L5‐S1 level. (C) Tumor base and its relation to the intervertebral foramen exposed entirely.

Under microscopic magnification, an encapsulated, rubbery, pinkish‐gray, pedunculated lesion was identified, with its base closely related to the L5–S1 neural foramen (Figure 3(C)). Careful dissection revealed extension of the tumor into the intervertebral foramen, with the L5 exiting nerve root attached to its caudal end (Figure 4).

**FIGURE 5:**
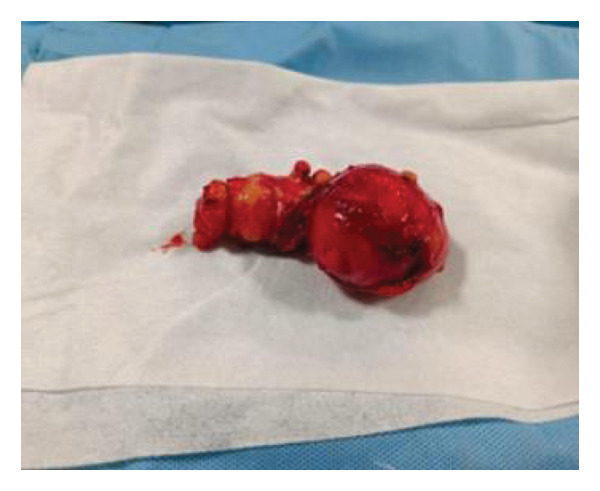
Excised tumor.

Further dissection through the intervertebral foramen allowed complete excision of the tumor along with its intact capsule (Figure 5). The involved L5 exiting nerve root was sacrificed to achieve complete tumor clearance. The excised specimen was sent for histopathological examination.

Meticulous hemostasis was achieved. A negative suction drain was placed, and the wound was closed in layers.

The procedure was uneventful. The patient was transferred to the neurosurgical intensive care unit for postoperative monitoring. No new immediate postoperative neurological deficits were observed other than mild weakness in dorsiflexion of the left great toe and reduced sensation on the lateral aspect of the left foot from ankle to little toe, which were present preoperatively.

Gross total (R0) resection of the nerve sheath tumor was achieved. The principles followed to ensure complete oncological clearance included the following:

1. Detailed preoperative imaging to assess the extent of the lesion.
2. Adequate exposure to visualize macroscopic tumor margins in all dimensions.
3. Maintenance of meticulous hemostasis throughout the procedure.
4. Use of magnification devices.
5. Capsule‐intact perineural dissection to preserve uninvolved nerve bundles.

Unplanned piecemeal excision without a multidisciplinary approach increases the risk of local recurrence, which may subsequently lead to malignant transformation, neurological deterioration, and increased morbidity and mortality.

Histopathology findings revealed hypercellular, hyalinized stroma with spindle cells having plump nuclei and hemorrhage with minimal thick‐walled blood vessels. Also, the lesion was surrounded by adipose tissue and nerve bundles, hence suggestive of a schwannoma.

**FIGURE 6:**
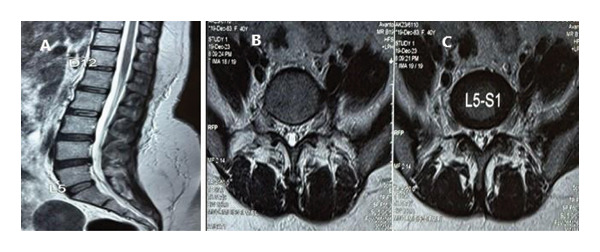
(A) Sagittal section. (B) Axial section. (C) Axial section postoperative images suggestive of no residual lesion.

The patient is periodically following up in the neurosurgery clinic. During the follow‐up period, a repeat MRI was performed at 6 months (Figure 6), which showed no radiological evidence of disease recurrence and the spine stability was maintained. She was under rehabilitation and showed marked improvement in the weakness of left great toe dorsiflexion from 4‐/5 to 4+/5, but the reduced sensation on the lateral aspect of the left foot persisted for 9 months of follow‐up.

## 3. Discussion

Nerve sheath tumors (schwannomas) arise at the Obersteiner–Redlich junction, the transition zone between oligodendrocytes and Schwann cells. They most commonly originate from the dorsal nerve roots and are usually intradural and extramedullary. Occasionally, they extend into the foraminal or extraforaminal regions, presenting as extradural lesions. Schwannomas may occur sporadically or in association with Neurofibromatosis type 2 or Carney complex [2].

Retroperitoneal extension of spinal schwannomas is rare, accounting for 3%–5% of cases. The large retroperitoneal space allows substantial tumor growth before symptom onset. Due to their slow progression and indolent nature, the retroperitoneal schwannomas present late or incidentally, with clinical features primarily dependent on tumor size and location [3]. Larger lesions may cause symptoms due to compression of adjacent neural, vascular, or visceral structures and may produce bony remodeling. Most spinal schwannomas are well‐encapsulated and nonadherent to surrounding tissues.

MRI is the diagnostic modality of choice and provides fundamental information for evaluating spinal tumors, guiding surgical planning through precise delineation of tumor extent and anatomical relationships. Schwannomas typically appear isointense on T1‐weighted images and hyperintense on T2‐weighted images. Cystic or necrotic changes may be present, in which case a preoperative biopsy may be required. Computed tomography (CT) aids in identifying bony erosion, foraminal widening, and calcifications and assists in surgical planning [4].

Complete tumor resection remains the definitive management for nerve sheath tumors and is associated with favorable clinical outcomes. Based on a systematic review and meta‐analysis conducted by Alvarez‐Crespo et al., it concluded that out of 2542 patients diagnosed with spinal schwannoma, 93.8% were treated with gross total resection and showed better prognosis and less chance of recurrence than subtotal resection [5]. Amongst the approaches, the traditional posterior, paraspinal, and anterior laparoscopic approaches are often constrained by technical challenges, prolonged operative duration, increased tissue disruption, greater blood loss, and elevated complication risk [3].

The conventional posterior transparaspinal approach (Table 1) is​ widely used for tumors located within the spinal canal or neural foramen. It is based on two principles. First, it involves incision of the paraspinal muscles, which may result in biomechanical compromise. Second, although it provides a surgical plane between the tumor capsule and adjacent neural and vascular structures, it is considered aggressive, as it often requires extensive laminectomy, splitting of the spinous process, possible pedicle violation, and facetectomy, resulting in significant muscle and bony disruption. Despite favorable oncological outcomes, this approach may lead to postoperative spinal instability, paraspinal muscle atrophy, and postspinal surgery syndrome. In addition, anterior retroperitoneal tumor extensions cannot be adequately addressed through a posterior route [6–8].

**TABLE 1:** Comparison between the various approaches in the management of a spinal schwannoma.

S. no	Parameter	Wiltse’s approach	Posterior transparaspinal approach	Backdoor anterolateral retroperitoneal approach
1	Surgical corridor	Paraspinal muscle‐splitting approach	Midline posterior with paraspinal muscle dissection	Lateral lumbar, muscle‐splitting, and retroperitoneal corridor
2	Muscle injury	Minimal	Significant due to extensive muscle dissection	Minimal or none
3	Bony resection	Minimal or none	Often required (laminectomy, facetectomy, and pedicle violation)	None
4	Visualization of neurovascular structures	Limited (dorsal root ganglion and vessels not well visualized)	Good visualization of intraspinal structures	Good visualization of extraforaminal and retroperitoneal structures
5	Access to the anterior retroperitoneal component	Poor	Not feasible	Excellent
6	Ability to achieve gross total resection in extraforaminal tumors	Limited	Possible but at the cost of extensive bony violation	High
7	Biomechanical preservation	Good	Poor	Excellent

Wiltse’s paraspinal approach (Table 1) was developed to minimize tissue injury by utilizing natural muscle planes and retraction systems. While it reduces muscle damage and provides adequate visualization for select lesions, it limits exposure of critical structures such as the dorsal root ganglion and adjacent vessels. Furthermore, this approach does not allow access to anterior retroperitoneal tumor extensions, as seen in the present case [9].

In contrast, the anterolateral retroperitoneal backdoor approach (Table 1) offers several advantages, such as direct access to the intervertebral foramen without the need for laminectomy or facetectomy, which are commonly required in posterior approaches. By avoiding posterior bony resection, this technique significantly reduces the risk of iatrogenic spinal instability and postoperative deformity, thereby eliminating the need for additional fusion procedures during tumor excision. Surgical morbidity is further minimized as paraspinal muscle dissection is avoided. Instead, the peritoneum is gently mobilized medially, while the psoas muscle and common iliac vessels are retracted laterally, creating a natural corridor to the L5–S1 foramen. Preservation of posterior musculoligamentous structures may result in reduced intraoperative blood loss, less postoperative pain, faster functional recovery, and shorter hospital stay [10].

Only a few case reports [11–14] have described the use of the anterolateral retroperitoneal approach for lateralized peripheral nerve sheath tumors (Table 2); therefore, this case report highlights the feasibility and uniqueness of this approach.

**TABLE 2:** Previous case reports regarding anterolateral retroperitoneal approach for paraspinal peripheral nerve sheath tumors.

References	Year	Tumor location	Gross total removal	Patient neurology	Postoperative neurology	Complication
Phan and Mobbs [10]	2016	Left EF of L5‐S1	Yes	Foot drop, dysesthesia	No aggravation	None
Shi et al. [11]	2016	PS of the lumbar	Yes	Back pain, dysesthesia	Improved	None
Boah and Perin [12]	2016	Left L4 nerve root	Yes	Radiculopathy	Full recovery	None
Rapport et al. [13]	2021	Right L1‐L2 foramen	Yes	Back pain, dysesthesia	Improved	None
Handa et al. [14]	2019	PS of the lumbar	No	Back pain	Improved	None

Hence, the spinal schwannomas exhibit unique diagnostic features, and the best treatment we can provide is surgical management. The selection of the most appropriate surgical approach significantly enhances the success of our treatment by means of providing complete surgical removal of the tumor without compromise to the spine stability. In our case report, we would like to highlight and conclude that for an extraforaminal spinal schwannoma extending retroperitoneally in the lumbosacral region, the minimally destructive backdoor anterolateral retroperitoneal approach aided us in preserving the spinal stability of the patient with maximal safe dissection, which was minimally disruptive in a more extensive classical posterior transparaspinal approach. We could also achieve total excision through the intervertebral foramen with a wider operative field in this approach, which was quite a challenge in the transparaspinal approach.

## Author Contributions

Kodeeswaran M. and Chiraag Hiran S. led the conceptualization, methodology, data curation, and project administration. Formal analysis involved Jagadish Chandrabose, Srikala Prasad, Priyadarshan K. P., Arun Narindar, Lakshmi Narasimhan Ranganathan, Bipin Chaurasia, and Ganesh P. S. Investigation and resource contribution were performed by Kodeeswaran M., Chiraag Hiran S., Srikala Prasad, Priyadarshan K. P., and Arun Narindar. The original draft was written by Chiraag Hiran S. under the supervision of Kodeeswaran M. All authors contributed to the final manuscript review and editing.

## Funding

The authors have nothing to report.

## Conflicts of Interest

The authors declare no conflicts of interest.

## Data Availability

The data that support the findings of this study are available from the corresponding author upon reasonable request.
